# Developing a Metric of Usable Space for Zoo Exhibits

**DOI:** 10.3389/fpsyg.2019.00791

**Published:** 2019-04-11

**Authors:** Heather Browning, Terry L. Maple

**Affiliations:** ^1^School of Philosophy, Australian National University, Acton, ACT, Australia; ^2^National Zoo and Aquarium, Yarralumla, ACT, Australia; ^3^Jacksonville Zoo and Gardens, Jacksonville, FL, United States

**Keywords:** usable space, exhibit, zoo, animal, welfare, wellness

## Abstract

The size of animal exhibits has important effects on their lives and welfare. However, most references to exhibit size only consider floor space and height dimensions, without considering the space afforded by usable features within the exhibit. In this paper, we develop two possible methods for measuring the usable space of zoo exhibits and apply these to a sample exhibit. Having a metric for usable space in place will provide a better reflection of the quality of different exhibits, and enhance comparisons between exhibits.

## Introduction

One of the most important environmental features for captive animals is the space they are provided. This was explored in Hediger’s pioneering work on zoo exhibit design (Hediger, 1950) and has been the focus of research and discussion since. Increased space can improve animal welfare through allowing for movement and exploration, expression of natural behaviors, room to provide a variety of exhibit furnishings, ability to make choices regarding social companions and environmental conditions, and for distance from public and group members as required.

Increased exhibit space for animals allows for performance of more natural behaviors and decreases occurrence of negative behaviors. Poor captive environments can cause welfare problems such as stereotypies and self-injurious behaviors (Goerke et al., 1987). Improving exhibit spaces helps relieve these problems as well as promoting positive behaviors, increasing overall behavioral repertoire (Hebert and Bard, 2000) and allowing for a fuller range of natural locomotive behaviors (Poole, 1991). An increase in usable space can also prompt an increase in activity, as adequate exercise can be difficult for animals held in small or simple environments (Hebert and Bard, 2000).

Exhibit size can also impact the social behavior of the occupants. Early hypotheses about overcrowding from decreased space leading to aggression were not borne out (de Waal, 1989; Miller et al., 2011). However, providing more space allows animals the ability to express their social preferences. Clark (2011) found that when a group of chimpanzees were given a new larger exhibit space, animals chose who to spend time near, with individuals seeming to spend more time near their “friends” (as evidenced by affiliative behaviors) and less time near those they disliked. Goerke et al. (1987) suggest that an increase in usable space may decrease social interactions overall; presumably because this is chosen by the animals. Supporting this, Kitchen and Martin (1996) found that common marmosets in larger cages spent less time in proximity to cage-mates, suggesting that in smaller cages, time spent in contact may not have been voluntary. The way the space is organized is also an important factor. Provision of areas to hide and escape can reduce aggression, and a simple increase in space alone may have little or no effect on rates of aggression within a group without attention to these factors (de Waal, 1989; Herrelko et al., 2015). Amount of space is still relevant, however, as more space allows for more provision of these features and, as indicated, also provides more choice as to proximity of social partners. Increase in usable space gives individuals more options for privacy and personal space, as well as the ability to provide more resources and decreasing competition for preferred areas (Hebert and Bard, 2000), all of which should provide social benefit to the exhibit occupants.

Beyond just the social benefits, availability of choice within their environment is of central importance to the welfare of captive animals. Increase in exhibit space can provide additional choice and control for animals. Coe et al. (2009) point out that increased usable three-dimensional space gives the animals more choice between different environmental gradients, such as light, temperature and humidity. Ross et al. (2009) suggest that increased space allows for spacing of preferred enclosure features, which can reduce competition for their use. Ross et al. (2011) found that gorillas and chimpanzees were highly selective of which space they used within their enclosure; indicating that they were making use of the choice available. As discussed above, space also allows animals to make social choices to meet or avoid one another when required. It can also allow animals to make the choice to avoid being too close to visitors when they find proximity distressing (see e.g., Hosey, 2005; Sherwen et al., 2015).

Exhibit size is thus valuable to animals for many reasons. Usually space is measured in terms of the floor space of the enclosure – a measurement in square meters that can be compared between exhibits. Guidelines and requirements for animal housing typically lay out space requirement in these terms (see for example EAPA, 2000). However, there is more to enclosure space than simply floor space. For example, consider two orangutan exhibits, both with the same size “footprint” in terms of floor space. One of these is a flat grassy exhibit, while the other contains a tall climbing structure of poles, ropes and platforms. As well as the obvious improvement in enclosure quality, this second exhibit also provides more space than the first for the orangutans to utilize for locomotion. The presence of additional exhibit furniture increases the internal space of the exhibit (Burton, 2004). Available exhibit spaces must be accessible to the animals, through the presence of furniture such as ropes, platforms and other pathways. These sorts of features “open up” the vertical space for use by the animal and increase total usable space. Many current enclosure modifications for arboreal animals, particularly primates, are centered around an increase in usable space through improving access to the vertical dimension (Anderson, 2014). Of central importance is ensuring that animals are able to use the space available to them. Quantity of space is generally less important for animal welfare than the complexity and usability of the space (Kitchen and Martin, 1996; Ross et al., 2011). By adding furniture which makes central and upper cage spaces accessible, this converts these areas into usable space and increases total availability (Maple and Perkins, 1995).

The importance of increasing usable vertical space for arboreal primates has been identified for decades (e.g., Maple, 1979, 1980) and is the focus of many recent enclosure modifications and studies. Historically, the vertical dimension was underutilized, as Maple and Finlay (1989) describe: “the last generation of captive environments for apes were deficient in providing for a vertical dimension of space. These generic ape grottos typically contained few climbing structures of insufficient height and complexity... the space available for locomotion is greatly expanded by building upward. Apes can use climbing structures to locomote through vertical space by brachiation or more cautious means” (1989, pp. 105–106). Increasing vertical space may be one of the best ways to improve the environments of great apes (Maple, 1979; Goerke et al., 1987), as well as other primates, allowing arboreal animals to display more of their natural behaviors. Orangutans, as the most arboreal of the great apes, have a particularly high requirement for vertical space (Maple, 1980). Hebert and Bard (2000) found that orangutans showed different behaviors at different heights within their enclosure; with more solitary and rest time in the higher strata, and more social and active time in the lower. They conclude that “usable space for orangutans is said to include adequate enclosure size as well as horizontal and vertical space” (2000, p. 249). Perkins (1992) found that orangutan activity level increased with enclosure size. Exhibit improvements for other primate species have had similar effects. Anderson (2014) examined the use of space by gibbons before and after the addition of hammocks, enrichment pulleys and log bridges to increase accessibility and create opportunity to use vertical space. This was successful, with the animals spending more time in the upper segments of the enclosure. Kitchen and Martin (1996) found that common marmosets showed increased activity and variety of behaviors in response to increases in enclosure size and complexity. Although most work so far has focussed on primates, increasing usable vertical space could also benefit other types of animals that are also vertically active, such as felids (Mellen and Sheperdson, 1997).

Volumetric space of this type can be described in a metric of usable space. The usable space of an enclosure includes not only the floor space, but all the exhibit features that the animals may use to move around and spend time on or in. It is a measure of the total usable surface area, or volume, that the animals can access. Maple and Finlay (1986) call for a measure of usable space that would allow unbiased comparison between complex zoo exhibits. There are many reasons to think that increasing usable space will benefit animal welfare, in terms of an expanded behavioral repertoire and an increase in social and environmental choice. Thus, a measure of usable exhibit space can stand in as a proxy measure for animal welfare and exhibit quality. Measurement of all usable exhibit features and the development of a function to combine these measures into a single “usable space” score would provide a valuable way of quantifying the space within exhibits and enabling a comparison between exhibits. This sort of measurement of spatial volume can provide a more meaningful index of space than simple exhibit size.

We differentiate here between usable space, as a measure of the potential space afforded by an exhibit in virtue of its design, and the actual use of space by its inhabitants, as shown by their behavior. Once usable space has been characterized by a metric such as the ones we provide, there is then a further question as to how the animals will use it. Not all usable space within an exhibit may be used by animals, for reasons of individual preference or temperament, but this does not mean that this should not count as usable space for the purposes of measurement. This distinction and its implications will be discussed in more detail further on. This paper will be concerned with usable space as a metric for evaluation of exhibits, rather than on the behavior of the animal inhabitants.

## Materials and Methods

**FIGURE 1:**
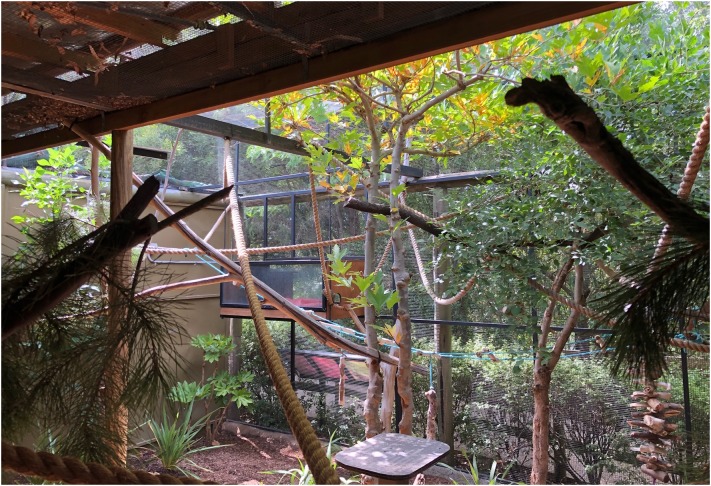
Pygmy marmoset exhibit at National Zoo and Aquarium.

The process of creating a usable space metric occurs in three parts: determining which exhibit features should be counted as part of the usable space, measuring these features and creating a formula that can combine the various measurements into a single metric that can be used to assess and compare enclosures. In the rest of the paper we will examine these steps through application to a real-world example, comparing two possible formulas that might be used in creating a usable space metric, before addressing some potential problems with the process and outlining its benefits. The exhibit chosen was the pygmy marmoset (*Cebuella pygmaea*) enclosure at the National Zoo and Aquarium in Canberra, Australia (Figure 1). This exhibit was chosen for ease of measurement, due to its small size, as well as the presence of complex vertical environmental features, which were necessary for best testing the calculations. As the animals were not present in the exhibit at the time of measurement, the study did not have the potential to impact animal welfare and as such no ethics approval was required, as per Australian National University and NHMRC guidelines.

### Determining Which Exhibit Features to Include

The first step in coming up with a measure of usable space, is deciding which exhibit features should be included within the measurement and calculation. As opposed to traditional measures of exhibit size, which simply take floor and wall dimensions, usable space measures will take into account all accessible exhibit features. General discussion of application of this method can be found in the discussion section. For this study, as the sample exhibit was for an arboreal primate the measured exhibit features incorporated the floor dimensions as well as all the vertical climbing surfaces. This included mesh cage sides and ceiling, ropes, poles, platforms and branches. Because of the small size of pygmy marmosets (body length around 15cm), all the smaller features of the exhibit (small branches, tree canopies) were considered usable space for these animals.

### Measurement

After identifying the relevant exhibit features to measure, the next step was to enter and measure them. Measurements for this exhibit were taken manually in cm, using a tape measure. Floor and wall dimensions were taken along edges; as were platform and nest box dimensions. One problem with measurements of usable surface area is calculations involving nearly one-dimensional linear pathways, such as ropes. Wilson (1982) came up with one solution, making measurements of such objects “as if the objects were flat planes” (1982, p. 204). It is unclear whether this meant using the diameter of the object as the flat surface dimension, or the circumference. In this work, we used the diameter for features the animals were likely to only use one side of (e.g., climbing along the top of ropes) and circumference for features they may use all sides of (e.g., climbing up and around poles). Trees, perches, ropes and poles were measured for length and circumference. For branching trees, all individual branches were measured, as these could all be individually occupied by the animals, due to their small size. Although branch circumferences varied slightly from base to tip, branches were treated as having a single circumference, taken near the middle of their length. Future work could look at validating this assumption through comparing calculations using this measurement to those using a more complex formula to account for circumference change along the branch length. Complex canopies of numerous small branches and leaves were too difficult to measure individually and were instead measured as though they were solid blocks, with their exterior dimensions being recorded. This approach could also be validated in future through comparison of this measurement to one more accurately recording the interior complexity of the canopies.

**FIGURE 2:**
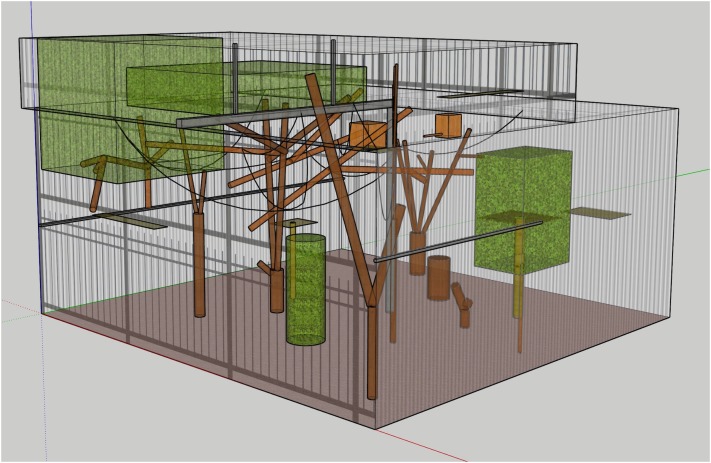
Digital 3D model of the pygmy marmoset exhibit.

Once all measurements were taken, these were then used to create a 3D model of the exhibit, using the program SketchUp Pro (2018 version) ^1^ (Figure 2). This model was extremely useful for visualizing different components of the exhibit and their relationship to one another, for taking any measurements that were missed in the initial exhibit measurement procedure, and for making volumetric space calculations (described in the next section).

### Creating a Formula for Combining Measurements Into a Usable Space Metric

Once measurements were obtained, the final step was to create a formula to combine these, to give us a single number representative of the usable exhibit space. For terrestrial animals that only move along flat spaces, this would be a straightforward sum of the various floor spaces they can use. For arboreal animals, this becomes more difficult with the addition of linear pathways. For aquatic and flying animals (possibly also some arboreal animals likely to leap and swing), there will be additional volumetric measurements of three-dimensional spaces the animals can move around within that also must be added to the model. There are two methods by which we think a useful measure of usable space can be obtained – that of total usable surface area, and usable volume, and both were tested for this exhibit.

#### Usable Surface Area

Total usable surface area (m^2^) is a sum of all the exhibit surfaces accessible to the animals – floor area, as well as platforms, ropes, mesh walls, exhibit furniture etc. (Wilson, 1982; Perkins, 1992; Lukas et al., 2003). For this exhibit, usable surface area was taken as the sum of surface areas of all the separate usable exhibit features. Flat surfaces, including floor, wall mesh, ceiling mesh and platforms, were calculated as the product of their side lengths. Other climbing structures, such as poles, ropes and branches, were calculated as the product of their length and their circumference. This treated the usable surface area for these objects as essentially the flattened surface area if they were to be rolled out. As the marmosets could move around any side of these features (e.g., climbing along top or bottom of ropes, or any side of a vertical perch), the entire surface area was considered usable. In some cases, a feature may not be considered usable on all sides (for example, if an animal could move along the top but not the bottom of a rope, as with an arboreal animal like a tree kangaroo). In these cases, the usable surface area would have to be modified accordingly, perhaps by taking the diameter of the object and treating it as an otherwise flat pathway of this width. For the pygmy marmosets there were no objects like this. As mentioned, since tree canopies were too complex to take the measurements of all the small branches within, usable surface area of these spaces was taken as the surface area around the edges of the canopy, as though it were a solid prism with external usable surface area. These separate surface area measures were then combined to form a total usable surface area score.

#### Usable Volume

Usable volume is a different type of measure, one which calculates how much of the total exhibit volume (m^3^) is accessible to the animals. Both Wilson (1982) and Perkins (1992) used a measure of exhibit volume, however this was not a measure of usable space as it was total exhibit volume and large parts of the total volume may be inaccessible to the animals. Ross et al. (2009) provided a useful way of thinking about usable enclosure volume. Their method was to divide the 3D space of the enclosure into blocks of 1 m^3^ and then score which of these blocks the animals are able to occupy, based on which exhibit structures are nearby; counting out “empty” spaces between exhibit features. Burton (2004) uses a similar method when running student courses on assessing animal exhibits – drawing up the enclosure as a 3D grid (in this case, usually 9 segments – low, middle, upper; left, center, right; front, middle, back).

**FIGURE 3:**
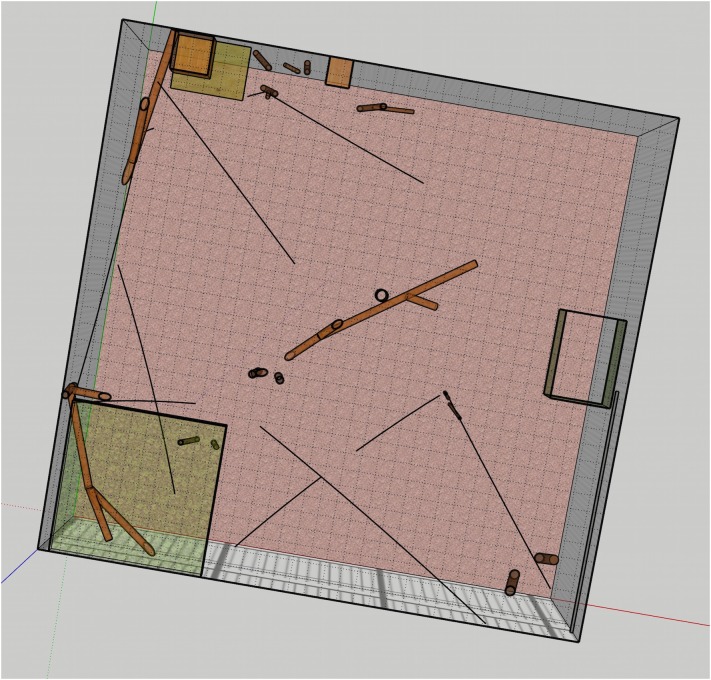
Cross-section of the exhibit model, with grid overlaid.

For the pygmy marmoset enclosure, usable volume calculations were taken by dividing the exhibit into many individual cubes, which were then scored for whether or not they could be used by the animal (i.e., whether exhibit features would allow the animals to access or use these spaces). Because of the small size of the animals, these cubes were taken as 15 cm × 15 cm × 15 cm (the body length of the marmosets). Essentially, the process involved dividing the enclosure into marmoset-sized boxes and counting those boxes which the marmosets could actually occupy. The 3D model produced in SketchUp made this process quite simple through the overlay of a grid onto the model. The model was then viewed as sections at each cut of 15 cm height (see Figure 3) and the number of boxes usable and not usable by the animals then individually counted through each section cut.

A cube was considered usable if it was adjacent to floor, mesh walls or ceiling, or if it contained a piece of exhibit furniture the animals were able to use. Usable volume did not include the usable spaces between objects that the monkeys could use to leap through; if these spaces were included as “usable” then the figure would be higher again. Where there was overlap between two different types of exhibit features within a single cube, this was only scored once. As well as being given a score for whether or not the cube was usable, it was also categorized according to which types of exhibit features it contained (floor, wall, ceiling, furniture). Due to substantial overlap between different objects (i.e., a single segment may have been made accessible to animals through both a branch and a rope), the general category “furniture” was used in calculations rather than specific sub-types of objects.

## Results

### Usable Surface Area

The results for the usable surface area calculations are presented at Table 1.

**Table 1:** Usable surface area calculations.

Feature	Surface area (m^2^)	Proportion of total
Mesh (walls/ceiling)	49.04	0.42
Floor	24.00	0.21
Platforms	1.51	0.01
Nest boxes	0.76	0.01
Poles	2.94	0.03
Trees/branches	35.10	0.30
- *Canopy*	26.96	0.18
- *Branches*	8.14	0.05
Ropes	2.79	0.02
Total	116.14	

The total usable surface area for this exhibit was found to be 116.14 m^2^. This is almost 5 times the space of the floor surface area alone, illustrating the difference between using this measure and traditional exhibit dimensions. This table also shows the total surface area sums for the different types of exhibit features (mesh, floor, platforms, nest boxes, poles, trees and branches, and ropes), as well as the proportion of the total contributed by each feature. These results show that by far the greatest proportion of the usable surface area (42%) is made up by the meshed walls and ceiling, results similar to those found by Wilson (1982). The next highest surface area (30%) was provided by trees and branches. The majority of this was accounted for by the canopies, as canopies made up 77% of the total surface area for this group. Floor space was the next highest (21%). Linear climbing structures such as ropes, poles and tree branches made up a very small proportion of the total surface area (10%), despite their large cumulative length (over 87 m).

### Usable Volume

Results for the usable volume calculations are shown at Table 2.

**Table 2:** Usable volume calculations.

Feature	Volume (m^3^)	Proportion of total
Floor	3.56	0.18
Mesh	6.94	0.35
- *Ceiling*	3.79	0.19
- *Walls*	3.15	0.16
Exhibit furniture	10.19	0.57
Total	20.14	

**Table 3:** Amount of volume overlap between exhibit features.

Features	Amount of overlap (segments)	Proportion of total
Floor and wall mesh	33	0.01
Wall and ceiling mesh	102	0.02
Floor and furniture	60	0.01
Walls and furniture	209	0.04
Ceiling and furniture	110	0.02
Total	514	0.10

The total usable volume for the exhibit was 20.14 m^3^. The total exhibit volume was calculated at 68.27 m^3^, so the usable volume represented around 30% of the total. This figure may seem low, but represents necessary empty space between exhibit features, both for keeper access and for animals to move around.

The proportions in Table 2 do not add up to 1, because they represent how many total segments each of these features types appear in and some of those segments have overlap between features, such as ceiling mesh and tree foliage. Table 3 shows the amount of overlap between different exhibit features.

This overlap contributes to around 10% of the total usable volume, which means that 90% of the volume is accounted for by segments consisting of only a single type of exhibit feature (in this case, counting all exhibit furniture as a single type of feature – the proportions would be much higher if broken down by branches, ropes etc.).

### Comparison

There are interesting differences between the breakdowns of the different measurement types, in terms of the contribution of the different types of exhibit features. While the usable surface area calculations showed 42% for meshed walls and ceiling, and 20% for floor; the usable volume showed 35% for meshed walls and ceiling, and 18% for floor. The usable surface area of exhibit furniture was only 37% of the total, while the usable volume provided by the furniture was 57% of the total. This suggests that the usable volume measure might be better at accounting for the contribution of exhibit furniture to usable space.

## Discussion

### Methods

In this paper, we designed and tested two different methods for quantifying the usable space of an exhibit. Both features shared the same first two steps – determining which exhibit features to include and taking measurements – and differed in the final step, of creating a formula through which to combine measurements in a single usable space metric.

Determining which exhibit features to include requires knowledge of the natural history of the species within the exhibit, as different exhibit features will count as usable or not usable, depending on the species. Which features are relevant will depend on the biology of the animals involved – the types of features which are usable for a wombat will be vastly different than those for a capuchin monkey, or an owl. Both the size of the animals and behavioral repertoire of the species will determine which exhibit features will be usable by the animals. There will also be an effect of the individual personalities and capacities of the animals held on which exhibit features can be used. Animals with physical limitations may not be able to access all features, while smaller or younger individuals may be able to access additional features. Individual behavior and preferences will also affect which features animals will choose to use, though this will be reflected more in actual space use than in the usable space features of the exhibit. Despite this, we can come up with a generalized list of those features which are likely to be important.

• Floor space –the basic floor space of an enclosure is a large part of the usable space for that exhibit. For strongly terrestrial animals, such as a kangaroo, this might still be the primary measure of usable space. For arboreal species, it will play less of a role. For raised or uneven surfaces, the surface area will be higher than the simple enclosure dimensions.
• Elevated platforms – the surface area of elevated platform spaces.
• Rocks – perhaps a type of elevated platform, the sitting and climbing surfaces of rocks count for those animals that can use them.
• Arboreal pathways – the length of ropes/logs/other pathways between elevated spaces.
• Climbing structures – the height (and possibly diameter/ circumference) of climbing poles/trees.
• Cage sides – for many primates and birds, the mesh of cage sides is usable space to move around on.
• Air volume – for flying (or leaping) animals, the total air volume of the enclosure could function as usable space for locomotion.
• Water volume – for aquatic animals, the volume of ponds and pools would count as usable space.
• Burrow volume – for burrowing animals, underground burrow systems would count as usable space.

Once the relevant features for any particular exhibit have been identified, they can then be measured and the total usable space calculated. Where there are areas in the enclosure that cannot be used because they are inaccessible to the animals though presence of barriers, or lack of accessible furniture, these should be subtracted from the total.

For pygmy marmosets, because of their small size and climbing ability, there were a very large number of separate usable exhibit features within their exhibit. For larger animals, or for animals with less agility, there may be fewer features included. This is an important step of the process for two reasons. First, because the accuracy of the usable space score will depend on inclusion of the right features – leaving out some usable features or including some inaccessible ones will give misleading scores. Second, in order to make comparisons between exhibits, the same types of features will need to be measured in each. There is a potential for future standardized lists of inclusions for each species to facilitate comparisons, but much is still likely to depend on individual discretion for each exhibit. A good understanding of the biology of the species in question will be crucial.

The second part of the process, measurement, took the most time. Manual measurement of all the individual usable exhibit features was time-consuming and labor-intensive. Measurement of straight floor and wall dimensions was relatively simple, but curved surfaces such as ropes and branches, were more difficult to measure accurately. Measurement of all the individual branches within the trees was the most intensive part of the process; though this would be easier for larger animals that would not separately use each of these small branches. In some cases it is likely to be impractical due to accessibility difficulties (not all tree spaces, for example, would be easy for a person to access and measure) as well as potential for inaccuracy. Once the measurements were taken, having the finished 3D model was useful for validating the accuracy of measurement through the depiction of exhibit features in relation to one another. Having such a model and would also be of use in the future for modeling potential changes to the exhibit.

For the measurement part of the process, a possible alternative method would be to create digital 3D exhibit models from which such measurements can be extracted. These can be created through a compilation of photographs (drone technology is particularly useful for gathering photographs from different heights and angles) or similar surveying methods (e.g., laser scan) through one of the many software programs available for such tasks – usually used in construction and engineering. Early attempts to use this procedure with photos of the pygmy marmoset exhibit were unsuccessful, with the models not stitching the photos together properly to create usable 3D models. However, this is a very promising area for future research, as use of this technology would significantly decrease measurement time, and increase accuracy, if used well; as it would combine both the measurement and model-creation into a single process, most of which would be done by the software rather than manually.

The final step was the calculation, and application of the two different formulas for quantifying usable exhibit space. Calculations of usable surface area did not require use of the 3D model, and were done easily within a spreadsheet containing the measurement data. Usable volume calculations were more complex, requiring first the building of the 3D model, and then manual counting of segments within the model. The same method could potentially be applied to counting segments through basic photos (ones not compiled into 3D models) or even visually assessing segments within the exhibit, but particularly at this scale, this would not be an accurate method. For large exhibits holding larger animals, where the scale of segmentation would be something more like 1 m^3^, these might be more useful methods.

Of the two methods, usable volume seems the most promising as a metric of usable exhibit space. It is better in accounting for the contribution of all exhibit features and more flexible in the types of exhibits it can score. As mentioned in the results, the usable volume calculation gave a much higher weighting for the effects of exhibit furniture in opening up usable space. This is because, although furniture such as ropes may not have much surface area, they have a large impact on how much of the exhibit they can make available to animals.

This method is also more flexible, able to provide scores for a range of exhibit types. As demonstrated here, it can account for usable space of complex vertical exhibits. Although the method was only applied to one type of exhibit – an arboreal primate – the results should generalize to any type of exhibits with usable volumetric space. These include those used by other arboreal or climbing species, exhibits with burrows or pools, aviaries and aquarium tanks. Future work applying these methods to a variety of exhibit types will help to refine the methods for different enclosure types. As usable volume seems the preferable method in most cases, through the rest of the paper discussion will be of this method only, though usable surface area calculations may still be valuable in making quicker judgements of usable exhibit space, or when dealing with terrestrial animals such as ungulates, on largely flat exhibits.

### Project Limitations

The primary limitation of the use of the usable volume calculation is that it may give misleading results in regards to the comparative assessment of enclosures. It does not necessarily contain all the information we require about exhibit quality and use. There will be cases in which a usable space score won’t accurately represent the actual use of exhibits by the animals, and also cases in which enclosures of lower overall quality are still given high usable space scores. However, these limitations seem possible to overcome.

One potential issue is that actual exhibit use by the animals may not reflect the usable space score. This follows the distinction we made in the beginning of the paper, between usable space as an exhibit metric, and space use as a behavioral measure of animals. While an exhibit might have a large usable space, the animals may in actuality only ever occupy a small portion of this space. In these cases, the usable space score will be misleading. This is likely to occur in cases where the space is undesirable to the animals, such as areas which are too open, or too close to the public. We certainly do not deny that use of space is important. As Kelling and Gaalema (2011) argue: “analysis of use of space is an essential element to link exhibit design and animal welfare” (2011, p. 602). If animals aren’t using portions of their exhibit, this may be reason to consider them not usable, or to try to find methods to make them more desirable. Ross et al. (2009) point out that studies of enclosure use help inform us about the preferences of the animals regarding the features of their available space, and can allow us to make modifications to encourage use of all areas. However, this is not the particular concern of this study: the usable space metric is not intended as a measure of actual enclosure use but of that space which is accessible by the animals and has the potential for use. Although actual use of space is important for animal managers and caregivers to pay attention to (not least because unused space is a waste of limited resources), it is not the focus here.

Additionally, a score of usable space may miss some important components of exhibit quality, particularly complexity. Environmental complexity has often been suggested as more important than exhibit space in terms of benefits to animals (Wilson, 1982; Goerke et al., 1987; Maple, 2007; Coe et al., 2009; Ross et al., 2011; Herrelko et al., 2015). However, this does not devalue the use of a usable space measure. Ross et al. (2011) point out that “these findings [regarding importance of spatial complexity over size] do not negate calls for larger spaces to improve captive wellbeing. Indeed, we are unaware of any reports that have empirically determined that providing too much space is detrimental to captive primate welfare” (2011, p. 203). Usable exhibit space and complexity will often be tightly connected, in both directions. Enclosure size affects the level of potential complexity – a larger enclosure has more space to add features which can increase complexity and use (Poole, 1991). As well, an increase in complexity will give an increase in usable space; and so usable space will reflect complexity as much as simply enclosure dimensions. There is the possibility of even constructing a score of exhibit complexity, as something like a ratio of total usable space to floor space. Much more so than traditional measures of enclosure size, usable space measures will give some representation of exhibit complexity.

Due to the nature of usable space calculations, a large but barren enclosure could still have a high usable space score while being low in quality. For example, when we include floor space (which we generally should, as it is a large part of the usable space), then one way of increasing the overall score is simply to add more floor space, without focussing on vertical complexity. For arboreal animals, such as orangutans, this seems like the wrong result, as elevated space is much more “usable” to them than ground space. This may simply be a separate issue of enclosure quality and provision of species-specific features (size isn’t everything, after all), but is certainly worth keeping in mind. There will, however, generally be overlap – the sorts of features which increase usable space, particularly vertically, will also be the sorts of features which increase environmental complexity. Where this is not the case, we need to keep in mind that while usable space is a useful metric for scoring and comparing different exhibits, it should not necessarily be used in isolation from other assessments of exhibit quality.

### Benefits of the Approach

There are several benefits to using the usable space metric developed in this paper. It allows for assessment of exhibit quality, comparisons between exhibits, assessment of potential exhibit improvements and the possibility to improve exhibit size guidelines and recommendations.

As discussed above, the usable space measure is not a perfect reflection of exhibit quality, as it does not entirely account for complexity, however, this measure will be closely linked with exhibit quality and certainly comes closer than existing basic measures of exhibit size. This method will also allow for comparisons between exhibits. Again, such comparisons are currently based either on basic exhibit size measurements, or on subjective assessments of how good or bad an exhibit seems to the observer. A usable space score provides an objective means of making more meaningful comparisons between exhibits. It must be kept in mind that such comparisons are only meaningful when comparing similar exhibits – those housing the same species (or species with the same requirements) and those for which the same sets of features have been measured and included in the score. There is no really meaningful way to perform an absolute comparison of, say, a Tasmanian devil and capuchin exhibit, except perhaps in regards to their relation to recommended or average usable space requirements for each species, as will be discussed further on.

Usable space calculations give us a means for assessing the benefits provided by possible exhibit improvements, as well as for coming up with the best ways to create improvements. By understanding the usable space calculation and which features contribute to it, we gain means to figure out how to increase the usable space of existing exhibits, or to build new exhibits that maximize usable space. One of the basic ways to increase usable space is still to increase exhibit size in terms of floor space. For entirely terrestrial animals, such as most hoofstock, this will be the primary method for increasing usable space. Another method for arboreal animals is through modifying walls or ceilings to allow for climbing – for example through use of mesh, hand-holds or cargo nets (Maple and Finlay, 1989). Mesh is often avoided, due to its unnatural appearance, but its contribution to usable space is important and methods of using climbable walls and ceilings while still maintaining a naturalistic appearance should be investigated. As seen in this study (and as found by Wilson, 1982), these factors can account for a high proportion of usable exhibit space and this means these values may represent the easiest way of increasing usable surface area within an exhibit. It is important again here to remember not to use usable space calculations without attending to the habits of the animals involved – for example, the pygmy marmoset exhibit showed 18% of the usable space as contributed by the floor area, but the monkeys rarely if ever use this space. Following Kitchen and Martin (1996) and Maple and Finlay (1989), finding ways of encouraging more use of this space – for example by providing woodchip for foraging – may help open up a lot of spatial opportunities for the animals.

Overall, it is likely to be more beneficial to increase usable space through increasing the complexity of an existing exhibit as opposed to replacing or upgrading. Due to space limitations within zoos, exhibit size will be restricted. It is, however, possible for zoos to increase the usable space available for animals by increasing use of the vertical dimension – adding platforms and pathways that create more spaces the animals are able to use and occupy. The exact methods used to increase usable space will depend on the particular exhibits and species, requiring the understanding of the species’ natural history as discussed in the previous section, but this measure allows for calculation of the change in usable space under different exhibit modifications and provides an excellent way of quantifying the value of such changes.

Finally, this work could have important implications for exhibit size recommendations. Although this project was not one of determining what the ideal recommended exhibit sizes for animals should be – rather of improving the ways in which we measure current exhibits – these measures could be useful in building future recommendations. Though we are able here to give a measure of usable space, this is not particularly meaningful without comparison to recommendations of ideal exhibit size. As current recommendations are usually based on floor space rather than more complex usable space, this will not give us much of a basis for determining whether exhibits are suitable. However, usable space recommendations could be incorporated into future exhibit recommendations and guidelines. Kelling and Gaalema (2011) note that there are not enough quantitative recommendations for exhibit design. Although there is a general consensus that there should be large and complex exhibits, this has not often translated into specific recommendations. Ross et al. (2011) make a similar point regarding “questions about if and how enclosure size for captive primates should be regulated. Currently, there is a tremendous range of enclosure size guidelines.... While each of these documents specifically notes the importance of other considerations such as vertical height and environmental complexity, it is clear that there is very little consensus on how much space is necessary to provide to this and other species. Given the push to formulate scientifically based management standards, further research that accounts for a range of environmental variables is necessary, especially studies that help elucidate the value of all the space that captive primates are not using” (2011, p. 206). It is our hope that having a measure of usable exhibit space might go some way toward being able to develop such guidelines, though it will take separate research to determine the usable space requirements for different species.

We can compare the measures found in this study to the traditional enclosure dimension measures to see their advantage. Usual space measures for this exhibit would simply represent the floor space (24 m^2^) and the exhibit height (3.05 m) without taking into account the use of these additional features. For example, the EAPA requirements for pygmy marmoset housing simply state that the animals require floor space of 2.5 m × 3.0 m (7.5 m^2^) and a height of 3 m (EAPA, 2000). Although reference is made to suitable provision of climbing structures, this is quantified by number of platforms and pathways rather than the space afforded by these. If we were to only consider floor surface area in this way, we would be underestimating the usable surface area of the exhibit by a factor of almost 5. Using the traditional methods, we could say that this marmoset exhibit exceeds minimum requirements by more than three times, but if we include all usable surface area this is much higher. Using the minimum EAPA requirements, we come to a required volume of 22.5 m^3^. The total exhibit volume was calculated at 68.27 m^3^, which is over three times the required space. Using the measure of usable volume, this comes out at 20.14 m^3^. This cannot be compared to the total required volume, as this volume would necessarily also include the empty space for keeper access and animal movement. Expression of exhibit requirements in terms of percentage of usable volume within the required space, or just an absolute value of minimum usable volume, would help capture this.

There are a number of methods by which usable space recommendations for exhibits could be developed, such as preferred social distance, animal body size, preference testing, and exhibit use studies (Innis et al., 1985; Petherick, 2007). Of some use may be information about home range or territory space in the wild, though zoos are often unlikely to have the resources to match this space, and as these can often reflect resource availability rather than space requirements *per se*, conclusions based on wild ranges may be misleading. Size requirements will depend on species-specific factors, as well as individual preferences of the animals involved. Preference tests can be a valuable tool in determining how much extra space is important to the animals – if the animals will work to gain access to extra space (as has been shown in studies on hens and rodents), then this space must be valuable to them; and at the point at which they would stop working for it, then it becomes welfare-neutral (Petherick, 2007). Finally, exhibit use studies can tell us how much of the space, and what type of space, the animals prefer to use, and can shed light on what would be an appropriate amount of usable space.

## Conclusion

There are many reasons to think that the size of exhibits provided for zoo animals will have important effects on their lives and welfare, through allowing more opportunities for choice and control, to exhibit natural behavior, and to maintain social groups. Currently, most exhibit size recommendations only refer to basic exhibit dimensions, without considering the space afforded by usable features within the exhibit. Here, we used measurements of a sample pygmy marmoset exhibit to develop two possible methods for measuring the usable space of zoo exhibits – usable surface area and usable volume. For arboreal species like the marmosets, usable volume calculations seem to better capture the contribution made to usable space by different exhibit features. Usable surface area calculations are simpler and could be applied to most solely terrestrial species. Having a measure of usable space in place will give a better indication of the quality of different exhibits, and allow for comparisons between exhibits. Use of digital methods for modeling and measuring exhibits may help make the process faster and more accurate, and this is a promising direction for future research. The introduction of the construct “wellness” suggests that future zoo exhibits will be aspirational rather than simply regulatory in their scope and function (Maple and Perdue, 2013). With an increasing focus on positive animal welfare, zoo professionals aim to ensure animals are thriving in their environment, as opposed to merely coping (Maple, 2014). Increasing usable space is one way to promote this end. If we try to arrange exhibit features to encourage thriving, exhibits will need sufficient size and complexity to achieve these results. Measurement of usable volumetric space will permit zoos to enhance wellness by attention to the details of space and the usable features within that space.

## Author Contributions

HB conceived the method, performed the measurements and calculations, and wrote the manuscript. TM contributed to the idea and planning, and helped to shape the manuscript.

## Conflict of Interest Statement

The authors declare that the research was conducted in the absence of any commercial or financial relationships that could be construed as a potential conflict of interest.

## Footnotes

1 www.sketchup.com/products/sketchup-pro
